# Blood Lead Levels and Potential Risk Factors for Lead Exposures Among South Asians in New York City

**DOI:** 10.1007/s10903-016-0403-5

**Published:** 2016-03-25

**Authors:** Paromita Hore, Munerah S. Ahmed, Slavenka Sedlar, Robert B. Saper, Deborah Nagin, Nancy Clark

**Affiliations:** 1 Bureau of Environmental Disease and Injury Prevention, New York City Department of Health and Mental Hygiene, 125 Worth Street, CN34C, New York, NY USA; 2 Boston University School of Medicine, Boston, MA USA

**Keywords:** Lead poisoning, South Asian, Ethnic disparities, Risk assessment, Biomonitoring

## Abstract

New York City’s South Asian children and pregnant women have a disproportionate burden of elevated blood lead levels. This study is the first to investigate blood lead levels and risk factors for lead exposures among South Asian New Yorkers. A survey and a finger-stick blood lead test using a portable analyzer were administered to 230 South Asian adults and children. Blood lead levels of 5 µg/dL or higher were found in 20 % of the adults and 15 % of the children, as compared to 5 % of adults and 2.5 % of children citywide. Factors associated with elevated blood lead levels were recent repair work at home, not speaking English, Bangladeshi or Indian ethnicity, and occupational risk factors. Public health professional should be aware that South Asians may be at an increased risk for elevated blood lead levels.

## Background

Lead poisoning is a preventable public health problem. Adverse health effects of elevated blood lead levels are well-documented [1, 2]. New York City (NYC) witnessed a dramatic decline in lead poisoning. Yet surveillance data indicates lead poisoning disproportionately affect minority and foreign-born populations, including children and pregnant women from Bangladesh, India, and Pakistan [3, 4].

In 2012, 14 % of NYC children with elevated blood lead levels were foreign-born with almost half born in Bangladesh or Pakistan. Similarly, 93 % of pregnant women with elevated blood lead levels were foreign-born with almost one out of four born in these countries. Surveillance data, however, is restricted to groups that receive routine blood lead testing. Blood lead testing in New York State (NYS) is mandatory for children ages 1 and 2 years and occupationally exposed adults. Older children and pregnant women are tested if providers assess them at risk for lead exposure. Consequently, information on the blood lead levels within South Asian communities is fragmented. In addition, the prevalence of risk factors and associations between these risk factors and blood lead levels is poorly understood.

Multiple factors may explain the increased vulnerability for lead poisoning among the city’s Bangladeshi, Indian, and Pakistani communities. Foreign birth or travel abroad may increase risk as environmental lead exposure may be more common in developing countries as compared to the United States (US) [5]. Use of imported products manufactured or obtained in countries where product safety standards may not be stringent can further contribute to their risk [6–8]. The NYC Department of Health and Mental Hygiene (DOHMH) investigated numerous cases of lead poisoning in South Asian children and adults associated with the use of products containing high levels of lead. Examples include: turmeric, chili powder, and other spices obtained in Bangladesh, India, or Pakistan; a variety of Ayurvedic and other traditional medicines both hand-made and manufactured in India; the eye cosmetics kohl, kajal or surma which are widely used in South Asia; and sindoor, a red or orange powder used for religious practices in Hinduism [7]. Despite these known vulnerabilities, little information is available about the blood lead levels of many segments of the South Asian population in the US, the prevalence of specific risk factors in these communities, and the association between these risk factors and blood lead levels. DOHMH conducted a study to understand these vulnerabilities by investigating blood lead levels and risk factors for lead exposures within NYC South Asian communities.

## Methods

### Study Design

This cross-sectional study consisted of a risk assessment survey and finger-stick blood lead test administered to a convenience sample of 230 NYC men, women, and children who self-identified as Bangladeshi, Indian, or Pakistani. The DOHMH Institutional Review Board approved the study.

### Recruitment and Data Collection

Recruitment and data collection occurred primarily in NYC’s South Asian neighborhoods from August 2008 to December 2009. DOHMH collaborated with two community-based organizations that provide a variety of services to these communities including English classes, support groups, and health education. DOHMH convened multilingual focus groups composed of women recruited by both organizations to review survey questions for clarity and cultural appropriateness, and to identify linguistically correct terms for certain risk factors. Focus group feedback and the community organizations guided recruitment strategies including the identification of screening locations. Community partners notified clients of screenings and distributed flyers prior to an event. In addition, project staff distributed flyers on the days of screening events. Screening locations included community and religious centers, schools, health clinics, and health fairs.

The survey consisted of questions on demographics, medical history, and environmental and behavioral risk factors for lead poisoning. In addition, participants were asked if they or anyone in the household performed certain jobs, hobbies, or activities associated with lead exposure. Bilingual DOHMH staff or, when necessary, trained volunteers administered the survey as a face-to-face interview. Nurses and phlebotomists collected blood samples using a portable ESA Magellan Biosciences CLIA-waived LeadCare II Blood Lead Analyzer with a reportable range from 3.3 to 65 μg/dL. Blood lead levels <3.3 μg/dL and >65 μg/dL were reported as “low” and “high”, respectively. Participants with finger-stick blood lead levels >8 μg/dL were offered confirmatory venous blood testing per NYS Department of Health requirements. Recommended quality control measures were taken during each screening event to ensure the accuracy of blood sample results.

### Statistical Analyses

Descriptive statistics analyzed socio-demographic characteristics, blood lead levels, and environmental and behavioral risk factors for lead poisoning. Associations were investigated using *t* tests, univariate regression analyses, and analysis of variance (ANOVA). Multivariate logistic regression modelling identified factors associated with blood lead levels of 5 μg/dL or greater. Environmental and behavioral risk factors were fitted in a multivariate logistic regression model. Age, gender and foreign birth status were a priori included into a separate multivariate logistic regression model together with socio-demographic variables associated with blood lead levels at a significance level of *p* < 0.20 in univariate analyses. A multivariate regression model with backward elimination that included these socio-demographic variables and environmental and behavioral variables was also tested. The natural logarithm of blood lead level was used in parametric analyses. Blood lead levels below the reportable range were substituted with the detection limit divided by the square root of two. Analyses were performed using SAS 9.1 and SPSS 19.

## Results

### Socio-demographic Characteristics

Participants were primarily adults (77 %) and females (60 %) (Table 1). The average age was 46.2 years for adults and 7.5 years for children. Most adults were foreign-born (99 %), whereas the majority of the children were born in the US (63 %). Approximately 30 % of participants have been in the US <5 years. Of the participants, 40 % identified as Indian, 35 % as Bangladeshi, 24 % as Pakistani, and 1 % as more than one South Asian ethnicity. The most frequent languages spoken among the 44 % who did not speak English were Bengali (34 %), Punjabi (34 %), Urdu (22 %), and Hindi (12 %). Higher education was reported by 47 % of adults. A household income below $20,000 was reported by 35 % of participants. Approximately 25 % of participants had no health insurance.

**Table 1:** Distribution of blood lead levels by socio-demographic, environmental and behavioral factors

Characteristics	*n* (%)	Geometric mean BLL (SD)^a^ (µg/dL)	BLL <5 (µg/dL)	BLL 5–<10 (µg/dL)	BLL ≥10 (µg/dL)
			*n* (%)	*n* (%)	*n* (%)
Total	230 (100)	3.3 (1.6)	187 (81)	34 (15)	9 (4)
*Socio-demographic factors*					
Age^b^					
Adult	178 (77)	3.3 (1.6)	143 (80)	26 (15)	9 (5)
Children	52 (23)	3.1 (1.5)	44 (85)	8 (15)	–
Gender					
Female	139 (60)	3.3 (1.7)	115 (83)	17 (12)	7 (5)
Male	91 (40)	3.3 (1.6)	72 (79)	17 (19)	2 (2)
Country of birth					
Foreign-born	195 (85)	3.3 (1.6)	157 (81)	29 (15)	9 (5)
Adults	176 (90)	3.3 (1.5)	–	–	–
Children	19 (10)	3 (1.6)	–	–	–
US-born	35 (15)	3.2 (1.5)	30 (86)	5 (14)	0 (0)
Adults	2 (6)				
Children	33 (94)				
Length of residence in the US (in years)^c,d^					
<5	71 (31)	3.5 (1.5)	55 (78)	15 (21)	1 (1)
5–9	64 (28)	3.4 (1.7)	52 (81)	9 (14)	3 (5)
10 or longer	95 (41)	3.1 (1.6)	80 (84)	10 (11)	5 (5)
Ethnicity^c,e^					
Bangladeshi	81 (35)	3.6 (1.7)	62 (77)	14 (16)	5 (6)
Indian	92 (40)	3.3 (1.6)	73 (79)	15 (16)	4 (4)
Pakistani	55 (24)	2.9 (1.4)	50 (91)	5 (9)	0 (0)
Other^f^	2 (1)		–	–	–
English language speaker^c^					
Yes	129 (56)	3.1 (1.7)	110 (85)	16 (12)	3 (2)
No	101 (44)	3.5 (1.6)	77 (76)	18 (18)	6 (6)
Education (adults)^c^					
Less than high school	33 (19)	3.3 (1.6)	26 (79)	5 (15)	2 (6)
High school	61 (34)	3.6 (1.8)	47 (77)	10 (16)	4 (7)
College or more	84 (47)	3.2 (1.6)	70 (83)	11 (13)	3 (4)
Household income					
<$20,000	80 (35)	3.3 (1.6)	63 (79)	14 (18)	3 (4)
$20,000–<$50,000	64 (28)	3.3 (1.7)	55 (86)	6 (9)	3 (5)
≥$50,000	23 (10)	3.3 (1.6)	18 (78)	5 (22)	0 (0)
Declined/don’t know	63 (27)	3.3 (1.6)	51 (81)	9 (14)	3 (5)
Had health insurance^c^					
Yes	177 (77)	3.2 (1.6)	149 (84)	22 (12)	6 (3)
No	53 (23)	3.7 (1.7)	38 (72)	12 (23)	3 (6)
*Environmental and behavioral factors*					
Number of risk factors reported					
≥1 risk factor	224 (97)	3.3 (1.6)	182 (81)	33 (15)	9 (4)
No risk factors	6 (3)	2.8 (1.4)	5 (83)	1 (17)	0 (0)
Repair work at home^c^					
Yes	64 (28)	3.9 (1.8)	43 (67)	17 (27)	4 (6)
No	166 (72)	3.1 (1.5)	144 (87)	17 (10)	5 (3)
Traveled outside of the US^c^					
Yes	115 (50)	3.5 (1.7)	88 (77)	20 (17)	7 (6)
No	115 (50)	3.1 (1.5)	99 (86)	14 (12)	2 (2)
Water damage at home^c^					
Yes	62 (27)	3.6 (1.8)	48 (77)	10 (16)	4 (6)
No	168 (73)	3.2 (1.6)	139 (83)	24 (14)	5 (3)
Occupational risks					
Yes	45 (20)	3.6 (1.6)	32 (71)	11 (24)	2 (4)
No	185 (80)	3.2 (1.6)	155 (84)	23 (12)	7 (4)
History of fractured bones					
Yes	11 (5)	3.0 (1.6)	9 (82)	2 (18)	0 (0)
No	219 (95)	3.3 (1.6)	178 (81)	32 (15)	9 (4)
Had Pica (ingested non-food items)					
Yes	26 (11)	3.3 (1.6)	22 (85)	4 (15)	0 (0)
No	204 (89)	3.3 (1.6)	165 (81)	30 (15)	9 (4)
Used imported ceramic ware^c^					
Yes	9 (4)	4.3 (1.7)	5 (56)	4 (44)	0 (0)
No	221 (96)	3.3 (1.6)	182 (82)	30 (14)	9 (4)
Used imported medicine products					
Yes	29 (13)	3.3 (1.5)	25 (86)	4 (14)	0 (0)
No	201 (87)	3.3 (1.6)	162 (81)	30 (15)	9 (4)
Used imported spices					
Yes	198 (86)	3.3 (1.6)	163 (82)	26 (13)	9 (5)
No	32 (14)	3.1 (1.5)	24 (75)	8 (25)	0 (0)
Used imported cosmetics					
Yes	22 (10)	3.3 (1.7)	19 (86)	1 (5)	2 (9)
No	208 (90)	3.3 (1.6)	168 (81)	33 (16)	7 (3)

^a^Log transformed finger-stick blood lead levels were used in all analyses

^b^Mean age for adults was 46.2 years (SD = 16.4), and for children <18 years mean was 7.5 years (SD = 4.8)

^c^This variable was found significant in univariate analyses and included in multivariate logistic regression models

^d^Mean length of residence in the US was 10.5 years (SD = 9.6)

^e^Post-hoc comparisons showed that the only significant difference in geometric mean blood lead level was between Pakistani and Bangladeshi

^f^Participants reported more than one South Asian ethnicity. The descriptive statistics were not calculated due to a small n. The two participants were excluded from multivariate logistic regression analyses

### Blood Lead Levels

Blood lead levels ranged from <3.3 to 29 μg/dL. The eight confirmatory venous blood samples were all within ±4 µg/dL of the corresponding finger-stick levels. Blood lead levels at or above 5 μg/dL, indicating elevated lead exposure, were found in 20 % of adults and 15 % of children (Table 1). Approximately 5 % of adults had levels of 10 µg/dL or greater, defined as lead poisoning by the NYC Health Code.

### Socio-demographic, Environmental, and Behavioral Risk Factors

In univariate analyses higher blood lead levels were associated with having no health insurance, not being an English speaker, having recent repair work or water damage at home, recently traveling abroad, ethnicity, education, use of imported ceramicware and length of residence in the US (Table 1). In a multivariate logistic regression model predicting blood lead levels of 5 µg/dL or greater, age, gender, foreign birth were included a priori, with other socio-demographic variables that were associated with blood lead levels in univariate analyses (ethnicity, language, health insurance status, education, and length of residence in the US). Only ethnicity remained a significant predictor of elevated blood lead levels. Bangladeshi participants were significantly more likely to have elevated blood lead levels than Pakistani participants (adjusted OR 3.58; 95 % CI 1.20–11.01) (Table 2). In a separate logistic regression model adjusted for all environmental and behavioral risk factors, recent repair work at home significantly increased the odds of having elevated blood lead levels (adjusted OR 3.22; 95 % CI 1.49–6.97) (Table 3). The observed effects of ethnicity and repair work at home remained statistically significant in a logistic regression model adjusted for the socio-demographic, environmental, and behavioral risk factors described above (Table 4). Logistic regression modelling with backward elimination resulted in four significant factors that increased odds of having elevated blood lead levels: recent repair work done at home (adjusted OR 4.54; 95 % CI 2.09–9.89); being of Bangladeshi (adjusted OR 5.19; 95 % CI 1.62–16.64) or Indian ethnicity (adjusted OR 4.90; 95 % CI 1.52–15.83); having had occupational risks (adjusted OR 2.69; 95 % CI 1.15–6.30); and being a non-English speaker (adjusted OR 2.27; 95 % CI 1.08–4.77) (Table 5). These factors remained significant when age, gender, and foreign birth were added to the model.

**Table 2:** Multivariate logistic regression analyses (socio-demographic variables)

Variables in the model	Adj. odds ratio (95 % CI)
Ethnicity (Bangladeshi)^a^	3.58 (1.20, 11.01)*
Ethnicity (Indian)^a^	3.01 (0.95, 9.48)
Non-English language speaker	1.66 (0.80, 3.47)
No Health Insurance	1.69 (0.75, 3.77)
Gender^b^	1.53 (0.74, 3.19)
Education (High school)^c^	1.51 (0.58, 3.94)
Foreign born	1.57 (0.38, 6.41)
Age (in years)	0.99 (0.97, 1.02)
Length of residence in the US (in years)	0.99 (0.95, 1.04)
Education (College)^c^	1.05 (0.39, 2.80)

* *p* < 0.05

^a^Reference group is ‘Pakistani’

^b^Reference group is ‘Female’

^c^Reference group is ‘Less than high school’

**Table 3:** Multivariate logistic regression analyses (behavioral and environmental variables)

Variables in the model	Adj. odds ratio (95 % CI)
Repair work at home^a^	3.22 (1.49, 6.97)**
Traveled outside the US^a^	1.67 (0.79, 3.52)
Used herbal medicine products^a^	0.54 (0.20, 1.46)
Occupational risks^a^	1.65 (0.71, 3.83)
Used glazed dishes^a^	2.11 (0.46, 9.64)
Pica^a^	0.57 (0.17, 1.88)
Used imported spices^a^	0.68 (0.26, 1.80)
Used imported cosmetics^a^	0.71 (0.19, 2.67)
History of broken bones^a^	0.75 (0.13, 4.14)
Water damage at home^a^	1.08 (0.47, 2.45)

** *p* < 0.01

^a^Reference group are those responding ‘No’ to a question about presence of a risk factor

**Table 4:** Multivariate logistic regression analyses (full model)

Variables in the model	Adj. odds ratio (95 % CI)
Repair work at home^a^	4.40 (1.80, 10.75)**
Ethnicity (Bangladeshi)^b^	5.28 (1.44, 19.41)*
Ethnicity (Indian)^b^	4.67 (1.31, 16.64)*
Non-English language speaker	2.03 (0.89, 4.61)
Used imported spices^a^	0.51 (0.17, 1.46)
Used herbal medicine products^a^	0.50 (0.17, 1.47)
No Health Insurance	1.69 (0.68, 4.22)
Occupational risks^a^	1.74 (0.66, 4.61)
Traveled outside the US^a^	1.59 (0.69, 3.64)
Used glazed dishes^a^	2.27 (0.41, 12.72)
Education (High school)^c^	1.57 (0.54, 4.62)
Pica^a^	0.61 (0.15, 2.44)
Gender^d^	1.33 (0.58, 3.09)
Education (College)^c^	0.73 (0.24, 2.25)
Foreign Born	1.53 (0.32, 7.23)
Water damage at home^a^	1.23 (0.47, 3.22)
Age (in years)	1.00 (0.97, 1.03)
Used imported cosmetics^a^	1.11 (0.26, 4.71)
Length of residence in the US (in years)	1.00 (0.95, 1.06)
History of broken bones^a^	0.90 (0.15, 5.31)

* *p* < 0.05; ** *p* < 0.01

^a^Reference group are those responding ‘No’ to a question about presence of a risk factor

^b^Reference group is ‘Pakistani’

^c^Reference group is ‘Less than high school’

^d^Reference group is ‘Female’

**Table 5:** Multivariate logistic regression analyses with backward elimination

Variables in the model	Adj. odds ratio (95 % CI)
Repair work at home^a^	4.54 (2.09, 9.89)***
Ethnicity (Bangladeshi)^b^	5.19 (1.62, 16.64)**
Ethnicity (Indian)^b^	4.90 (1.52, 15.83)**
Occupational risks^a^	2.69 (1.15, 6.30)*
Non-English language speaker	2.27 (1.08, 4.77)*

* *p* < 0.05; ** *p* < 0.01; *** *p* < 0.001

^a^Reference group are those responding ‘No’ to a question about presence of a risk factor

^b^Reference group is ‘Pakistani’

## Discussion

The study is the first, to our knowledge, to examine blood lead levels and potential risk factors within NYC’s South Asian communities and to include adults with and without occupational exposures, non-pregnant and pregnant women, and younger and older children. Understanding lead exposure risks among these communities is necessary to address and reduce disparities in blood lead levels.

One of our main findings was the higher risk for elevated blood lead levels in this cohort. This study not only presented an indication of the blood lead levels within this at-risk group, it also helped identify individuals with elevated blood lead levels that may not have been otherwise captured. Blood lead levels of 10 µg/dL or greater were found in 5 % of adult participants. This was considerably higher than findings from the 2004 NYC Health and Nutrition Examination Survey (CHANES) which estimated 0.5 % of the city’s adult population to have such levels [9]. Elevated blood lead levels of 5 µg/dL or greater were found in 20 % of adult participants—four times higher than the CHANES findings. Similarly, 15 % of child participants had blood lead levels of 5 μg/dL or higher compared to 2.5 % estimated among all NYC children [4].

Recent repair work at home and occupational risk factors were significantly associated with blood lead levels. The health impact of work that disturbs lead-based paint is well documented [10, 11]. Implementing safe work practices is necessary for minimizing lead exposures for both residents and workers. Engaging in occupations or hobbies with lead exposure risks such as construction, bridge/steel work, target shooting, or metal or battery recycling was reported by 20 % of participants. Almost 30 % of participants who reported occupational risks had blood lead levels of 5 μg/dL or higher. Interestingly, Punjabi was the most frequent language spoken by participants reporting occupational risk factors suggesting Punjabi speakers may be at higher risk for occupational lead exposure. This also suggests prevalence of certain risk factors varies within sub-groups of the city’s South Asian communities.

Differences in blood lead levels between ethnicities further indicate that South Asian communities are not uniformly at risk. Bangladeshi or Indian participants had significantly higher blood lead levels compared to Pakistani participants; however, we cannot draw conclusions as to reasons for these differences. Significantly increased odds of having elevated blood lead levels were also found among non-English speakers who are more likely to be foreign-born [12] with higher blood lead levels probably due to both recent and past exposures [13].

Among other risk factors associated with blood lead levels was lack of health insurance. Approximately 30 % of participants without health insurance had blood lead levels 5 µg/dL or higher. Individuals without insurance may delay or forego accessing health care services and have poorer health outcomes [14–18]. Minority and low-income communities may not only be disproportionally exposed to environmental contaminants but also fare worse to such exposures due, in part, to difficulties in accessing health care [19, 20]. Having health insurance may serve as a protective factor in another way. Focus group members reported a preference for using allopathic medicines because they are covered by health insurance as opposed to Ayurvedic medications and supplements, some of which have been found to contain high levels of lead [6, 7].

Recent travel outside the US was also associated with higher blood lead levels. Unsurprisingly, Bangladesh, India, and Pakistan were the most frequently visited countries. The ease of modern travel has led to an increase of transnational communities–communities of migrants that often live in two countries, the country of migration and the ancestral country [21]. Migrants may spend prolonged periods in developing countries where exposures to higher levels of environmental lead are likely [22, 23]. Travel also increases access to less regulated consumer products which may contain high levels of lead. Users may obtain these contaminated products overseas, either through self-purchase while travelling or from relatives, friends, or providers who live abroad. Access to global markets due to rapid transport options allows such products to also be available for purchase in US markets or online [24, 25].

In this study, there were factors which may have contributed to the observed prevalence seen for certain risk factors. For example, only 13 % of participants reported using non-allopathic medicines although such products are widely used in South Asia [26]. Several factors may have contributed to this difference. The study involved a convenience sample of South Asians residing in NYC. South Asian immigrants are likely to differ from those living in South Asia given the very nature of the immigrant experience. In addition, in many South Asian countries access to healthcare is limited because of costs and geography. This may not be the case in NYC as affordable healthcare is more readily accessed. As mentioned above, participants in our focus groups expressed preference for using allopathic medicines because they are covered by health insurance which may explain such low prevalence of use. In addition, some participants may have been reluctant to disclose use of such products during the survey. This reluctance was seen among participants using imported medicine products. More than half of the users reported not discussing their use with a medical provider. The main reasons for not discussing use were: “It was not important for the provider to know”, “The provider never asked about it” and “The provider would not understand or approve.”

While DOHMH continues to identify lead poisoning cases associated with use of consumer products such as imported spices, cosmetics, and medicines or remedies among NYC’s South Asians [7], in this study a significant association was not observed between use of such products and blood lead levels. Several limitations may have contributed to the lack of significance for these and other risk factors. A small sample size may have limited the power to detect significant associations. Another limitation was the instrument detection limit of 3.3 µg/dL, which was considerably higher than the geometric mean blood lead level of 1.79 µg/dL estimated by CHANES [9]. The imputation method used in this study for values below the detection limit, while common, may have over- or underestimated the mean blood lead levels.

Despite these limitations, the study had several strengths. One strength was the unique approach and flexibility provided by the sampling design. The portable diagnostic device and conducting screenings in familiar and convenient facilities during business and non-business hours encouraged recruitment. Staff members knowledgeable with participants’ cultures and languages were able to effectively probe for specific information during interviews. Partnerships with trusted and respected community organizations greatly fostered support for and participation in this study. These organizations continue to serve as conduits in the dissemination of health prevention messages.

Our approach can easily be adapted to other vulnerable populations to investigate potential lead exposure risks, develop interventions, and disseminate health messages. Such an approach can identify subsets of a population with increased vulnerabilities. For example, our study showed that non-English speakers were at a significantly higher risk for elevated blood lead levels. Approximately one out of four non-English speakers had blood lead levels of 5 µg/dL or higher. Languages spoken by these participants were Bengali, Hindi, Urdu, and Punjabi. Punjabi was a language for which DOHMH previously did not have educational materials. Consequently, outreach materials were translated into Punjabi to supplement existing DOHMH literature. These materials continue to be disseminated by DOHMH and partner organizations. Language is a critical component of risk communication for developing effective public health interventions. These findings can help guide risk assessment and the development of lead poisoning prevention strategies for at-risk populations.

## Conclusions

Study findings clearly suggest South Asians are at an increased risk for lead exposure as 20 % of adults—four times the CHANES finding, and 15 % of children compared to 2.5 % estimated among all NYC children, had a blood lead level of 5 µg/dL or higher. Factors including recent repair work at home, not speaking English, Bangladeshi or Indian ethnicity, and occupational risks were significantly associated with elevated blood lead levels. Public health professionals, medical providers, and community organizations should be aware that South Asians may be at an increased risk for elevated blood lead levels and should screen South Asians particularly those with these risk factors. To reduce the disparity in lead exposures between South Asians and the general population, greater efforts to identify, remove, and raise awareness about lead hazards common in these communities are needed.
